# Overinterpretation is common in pathological diagnosis of appendix cancer during patient referral for oncologic care

**DOI:** 10.1371/journal.pone.0179216

**Published:** 2017-06-07

**Authors:** Mark A. Valasek, Irene Thung, Esha Gollapalle, Alexey A. Hodkoff, Kaitlyn J. Kelly, Joel M. Baumgartner, Vera Vavinskaya, Grace Y. Lin, Ann P. Tipps, Mojgan V. Hosseini, Andrew M. Lowy

**Affiliations:** 1 Department of Pathology, Division of Anatomic Pathology, University of California San Diego Medical Center, San Diego, California, United States of America; 2 Department of Surgery, Division of Surgical Oncology, University of California San Diego Moores Cancer Center, La Jolla, California, United States of America; Duke University, UNITED STATES

## Abstract

**Context:**

Low-grade appendiceal mucinous neoplasm (LAMN) and appendiceal adenocarcinoma are known to cause the majority of pseudomyxoma peritonei (PMP, i.e. mucinous ascites); however, recognition and proper classification of these neoplasms can be difficult despite established diagnostic criteria.

**Objective:**

To determine the pathological diagnostic concordance for appendix neoplasia and related lesions during patient referral to an academic medical center specialized in treating patients with PMP.

**Design:**

The anatomic pathology laboratory information system was searched to identify cases over a two-year period containing appendix specimens with mucinous neoplasia evaluated by an outside pathology group and by in-house slide review at a single large academic medical center during patient referral.

**Results:**

161 cases containing appendix specimens were identified over this period. Forty-six of 161 cases (28.6%) contained appendiceal primary neoplasia or lesions. Of these, the originating pathologist diagnosed 23 cases (50%) as adenocarcinoma and 23 cases (50%) as LAMN; however, the reference pathologist diagnosed 29 cases (63.0%) as LAMN, 13 cases (28.3%) as adenocarcinoma, and 4 cases (8.7%) as ruptured simple mucocele. Importantly, for cases in which the originating pathologist rendered a diagnosis of adenocarcinoma, the reference pathologist rendered a diagnosis of adenocarcinoma (56.5%, 13 of 23), LAMN (39.1%, 9 of 23), or simple mucocele (4.3%, 1 of 23). The overall diagnostic concordance rate for these major classifications was 71.7% (33 of 46) with an unweighted observed kappa value of 0.48 (95% CI, 0.27–0.69), consistent with moderate interobserver agreement. All of the observed discordance (28.3%) for major classifications could be attributed to over-interpretation. In addition, the majority of LAMN cases (65.5%) had potential diagnostic deficiencies including over-interpretation as adenocarcinoma and lacking or discordant risk stratification (i.e. documentation of extra-appendiceal neoplastic epithelium).

**Conclusions:**

Appendiceal mucinous lesions remain a difficult area for appropriate pathological classification with substantial discordance due to over-interpretation in this study. The findings highlight the critical need for recognition and application of diagnostic criteria regarding these tumors. Recently published consensus guidelines and a checklist provided herein may help facilitate improvement of diagnostic concordance and thereby reduce over-interpretation and potential overtreatment. Further studies are needed to determine the extent of this phenomenon and its potential clinical impact.

## Introduction

Mucinous appendiceal neoplasms that may give rise to pseudomyxoma peritonei (PMP) remain a challenging tumor type for pathological classification despite their description dating back to the 19^th^ century when the term PMP was first used by Werth.[1] Mucinous appendiceal neoplasms comprise a spectrum from low-grade appendiceal neoplasm (LAMN) to various grades of invasive adenocarcinoma.[2]

PMP is clinically defined as mucinous or gelatinous ascites and peritoneal implants associated with the pathological finding of neoplastic mucin-producing cells in the peritoneal cavity.[2,3] It is now understood that the vast majority of PMP arise from perforated mucinous neoplasms of the appendix, and represent their peritoneal extension, with a minority of cases being due to mucinous adenocarcinomas arising from other sites.[4] Ovarian origin of PMP is rare and generally associated with mucinous adenocarcinoma arising from mature cystic teratoma.[5,6] Most cases of PMP have low-grade histologic features (sometimes termed “low-grade mucinous carcinoma peritonei” according to the World Health Organization (WHO)), while close to one quarter of cases have high-grade histologic features (sometimes termed “high-grade mucinous carcinoma peritonei” according to the WHO) with a corresponding worse prognosis.[4] In addition, the presence of signet ring cells has emerged as an independent negative prognostic factor, especially if tissue invasion is seen.[7,8]

Although appendiceal neoplasms are relatively uncommon and encountered in less than 1–2% of incidental appendectomies, mucinous neoplasms and well-differentiated neuroendocrine tumors, also known as “carcinoids”, comprise the majority. [9,10] In contrast to neoplasia, by far the most common appendiceal diagnosis in surgical pathology is acute appendicitis.[11,12]

Several classification schemes have been published to help determine risk for recurrent disease in LAMN.[3,13–17] Studies have demonstrated that patients with LAMNs that are confined to the appendix remain disease free upon follow up.[14,15,18,19] Additionally, the presence of acellular mucin or neoplastic epithelium at the appendectomy margin of LAMNs confined to the appendix is not a predictor of peritoneal recurrence.[15,19] Moreover, a clear link has been established between the presence of extra-appendiceal neoplastic epithelium and risk for recurrent disease, wherein LAMNs lacking extra-appendiceal neoplastic epithelium are considered to be at low risk of peritoneal recurrence and LAMNs with extra-appendiceal neoplastic epithelium are at high risk of recurrence.[14,15,18,20]

Clinical management of LAMN with and without extra-appendiceal neoplastic epithelium is controversial.[17,21] A diagnosis of LAMN with low risk of recurrence can thus be used to justify conservative clinical follow-up, while at some centers LAMN with high risk of recurrence has been used to justify the pursuit of more aggressive therapies such as right hemicolectomy, cytoreductive surgery (CRS), with or without hyperthermic intraperitoneal chemotherapy (HIPEC) upon identification of dissemination to the peritoneal surfaces.[18] Other institutions have reported successful long-term outcomes with close observation of select patients with LAMN with high risk of recurrence, including some patients with limited peritoneal dissemination.[22] Our institution has adopted an individualized approach that, in general, includes close observation of patients with LAMN with low or high risk of recurrence, in the absence of peritoneal dissemination.

Because proper histologic classification can strongly influence both prognosis and treatment,[23] we performed a retrospective pathological chart review to measure the actual concordance rate between community pathologists and a single large academic medical center during patient referral.

## Materials and methods

### Human research protections program

This study was approved by the Institutional Review Board Human Research Protections Program at the University of California San Diego (UCSD). Informed consent was not required for this study.

### Identification of appendix cases

Retrospective chart review was performed for a 2-year period (2014–2015) and included pathology records obtained from the anatomic pathology laboratory information system (AP LIS) and the general electronic medical record. The AP LIS was searched to identify pathology cases containing appendix resection specimens (i.e. appendectomies, right hemicolectomies, etc.) with mucinous neoplasia evaluated both by an outside pathology group and by in-house slide review at UCSD during patient referral. Cases were included if there was a primary appendiceal neoplasm or lesion, and excluded if incidental or contained secondary neoplasia or other unrelated pathology. In addition, pathology records had to be readily available at the time of chart review.

### Pathologic evaluation and concordance

A retrospective chart review of original pathology reports was performed for all cases. Prospective pathological evaluation at the reference institution, a single large academic medical center (UCSD), was performed solely by a subspecialized GI pathology clinical service via protocol in-house slide review during patient referral for oncologic care. None of the cases were secondary consultations specifically requested by the original evaluating pathologist. Reference pathologists were not provided with prior study specific training sets. The histopathological slides were reviewed prospectively in 91% of the overall cases by at least one fellowship-trained GI pathologist and 74% by a GI pathologist with special interest in appendiceal neoplasia (MV). All cases having disagreement with the originating pathologist were evaluated prospectively by the lead study pathologist (MV) and shown to more than one reference pathologist. In general, such cases are returned to the originating institution after a short grace period; therefore, a comprehensive retrospective histopathological review was not performed. Example microscopic images from selected available cases are provided for illustration.

Concordance was measured as the total number of cases placed in the same diagnostic category (LAMN, adenocarcinoma, mucocele) by both the initial pathologist report and the second evaluation report. Cases were placed in the adenocarcinoma category if the term “adenocarcinoma” was included in the final pathological diagnosis. For cases without adenocarcinoma in the final pathological diagnosis, utilization of the term LAMN or other borderline malignant terminology such as “uncertain malignant potential” was placed the case in the LAMN category. “High-risk” LAMN refers to the presence of extra-appendiceal neoplastic epithelium which confers a high-risk of disease recurrence in the peritoneum, whereas “low-risk” LAMN here refers to the lack of extra-appendiceal neoplastic epithelium and includes cases with only extra-appendiceal acellular mucin and also cases thought to be entirely confined to the appendix [15]. Cases in which the location of the neoplastic epithelium could not be definitively determined were categorized as ambiguous, and cases lacking documentation of the presence or absence of extra-appendiceal neoplastic epithelium on the original pathology report were noted. In addition, mucocele as used herein refers to a non-neoplastic simple mucocele (sometimes also called a “mucous retention cyst”). Definitions of over-interpretation and under-interpretation are utilized as described previously [24]. Briefly, over-interpretation is defined as reporting a diagnosis of a higher category of malignancy than the reference; whereas, under-interpretation is defined as reporting a diagnosis of a lower category of malignancy than the reference. Unweighted Kappa values to determine interobserver agreement were calculated according to Fleiss and Cohen.[25]

## Results

### Overall case and originating practice characteristics

161 cases were identified that included appendix specimens over the period of study. Forty-six of 161 cases (28.6%) contained appendiceal primary neoplasia or lesions. Of the 46 initial diagnostic case surgical specimens, 35 (76.1%) were appendectomies, 6 (13.0%) were right hemicolectomies, 2 (4.3%) were appendectomies with partial cecectomy, 1 (2.2%) was an ileocecectomy, 1 (2.2%) was an appendectomy with cecectomy and pelvis mass excision, and 1 (2.2%) was an appendectomy with hysterectomy, bilateral salpingo-oopherectomy, splenectomy, and omentectomy. The 115 excluded cases had a wide variety of diagnoses including incidental appendectomies, secondary involvement by other neoplasms including mesothelioma, leiomyosarcoma, serous ovarian carcinoma, and other diagnoses unrelated to primary appendiceal disease. No cases were excluded due to lack of availability of pathology records. Patients referred for primary appendiceal neoplasia ranged in age from 29 to 81 years old with an average of 55 years old (median 59 years old) and patients were more likely to be female (M:F ratio 1:1.56). The in-house slide review was generally requested by an attending surgical oncologist during patient referral for oncologic care. The originating pathologist practices were usually mid-sized (number of pathologists, average 10, median 7) and non-academic (91.3%) with moderate surgical case volumes (average 22,000 accessions per annum, median 18,000 accessions per annum). The primary signing pathologist was usually not subspecialized in gastrointestinal pathology (87%) and consulted another pathologist(s) in a large fraction of the cases (37%).

### Pathological evaluations and diagnostic concordance

**Fig 1:**
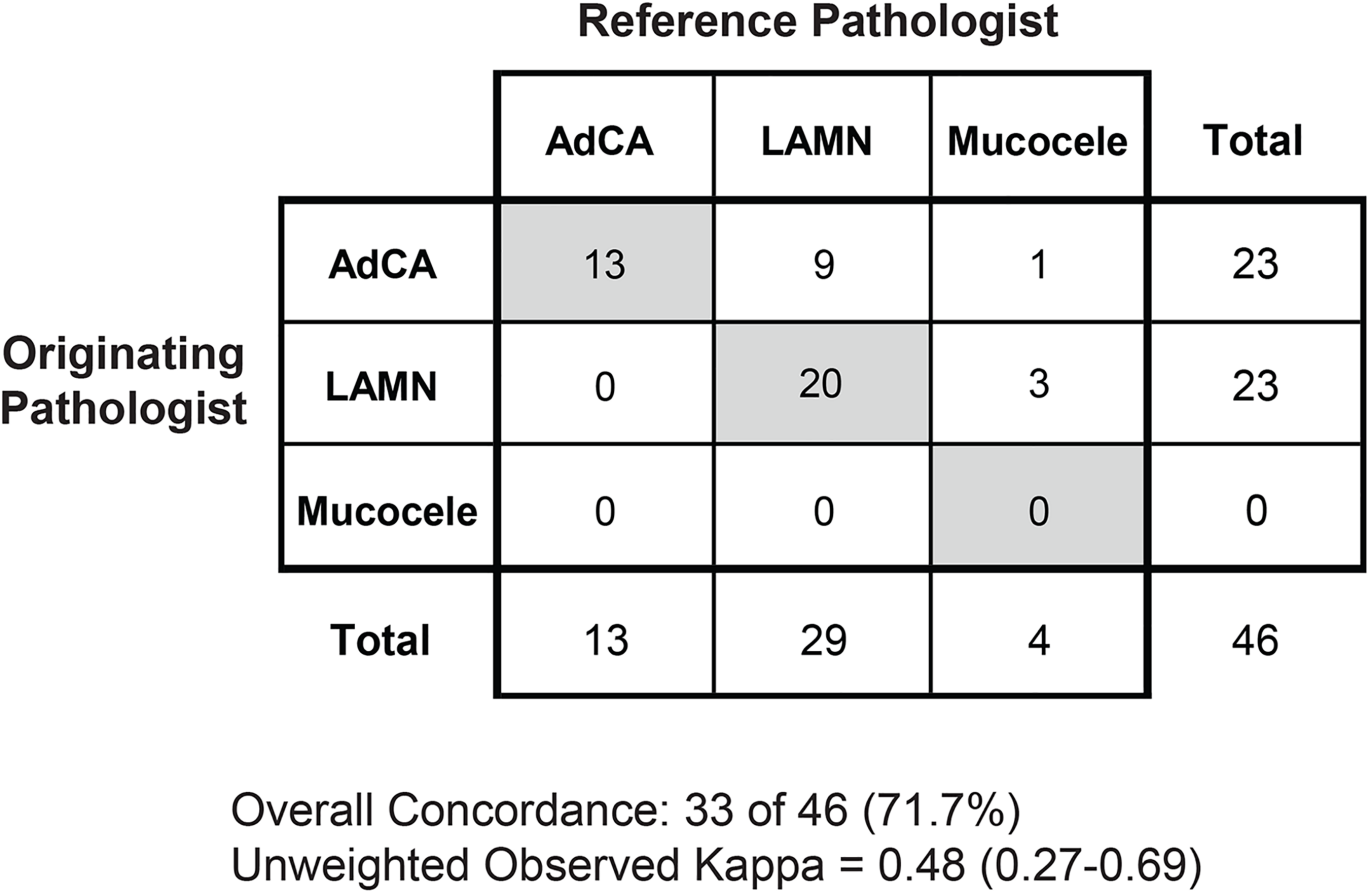
Concordance for major diagnostic categories of appendiceal lesions. Greyed boxes indicate concordant diagnoses between the originating pathologist and academic pathologist. AdCA indicates adenocarcinoma; LAMN, low-grade appendiceal mucinous neoplasm.

The distribution of appendiceal diagnoses from the originating pathologist was 50% adenocarcinoma (23 of 46), 50% LAMN (23 of 46), and 0% mucocele (0 of 46, Fig 1). In contrast, the distribution of appendiceal diagnoses from the reference academic pathologist was 28.3% adenocarcinoma (13 0f 46), 63% LAMN (29 of 46), and 8.7% mucocele (4 of 46). Subclassification demonstrated that the adenocarcinomas were 30.8% (4 of 13) adenocarcinoma ex goblet cell carcinoid, 23.1% (3 of 13) adenocarcinomas arising from low-grade appendiceal mucinous neoplasm (LAMN), and 46.2% (6 of 13) mucinous adenocarcinomas not otherwise specified. LAMNs were predominantly high-risk with extra-appendical neoplastic epithelium (62.1%, 18 of 29), low-risk without extra-appendiceal neoplastic epithelium (24.1%, 7 of 29), or not specified or ambiguous (13.8%, 4 of 29).

**Fig 2:**
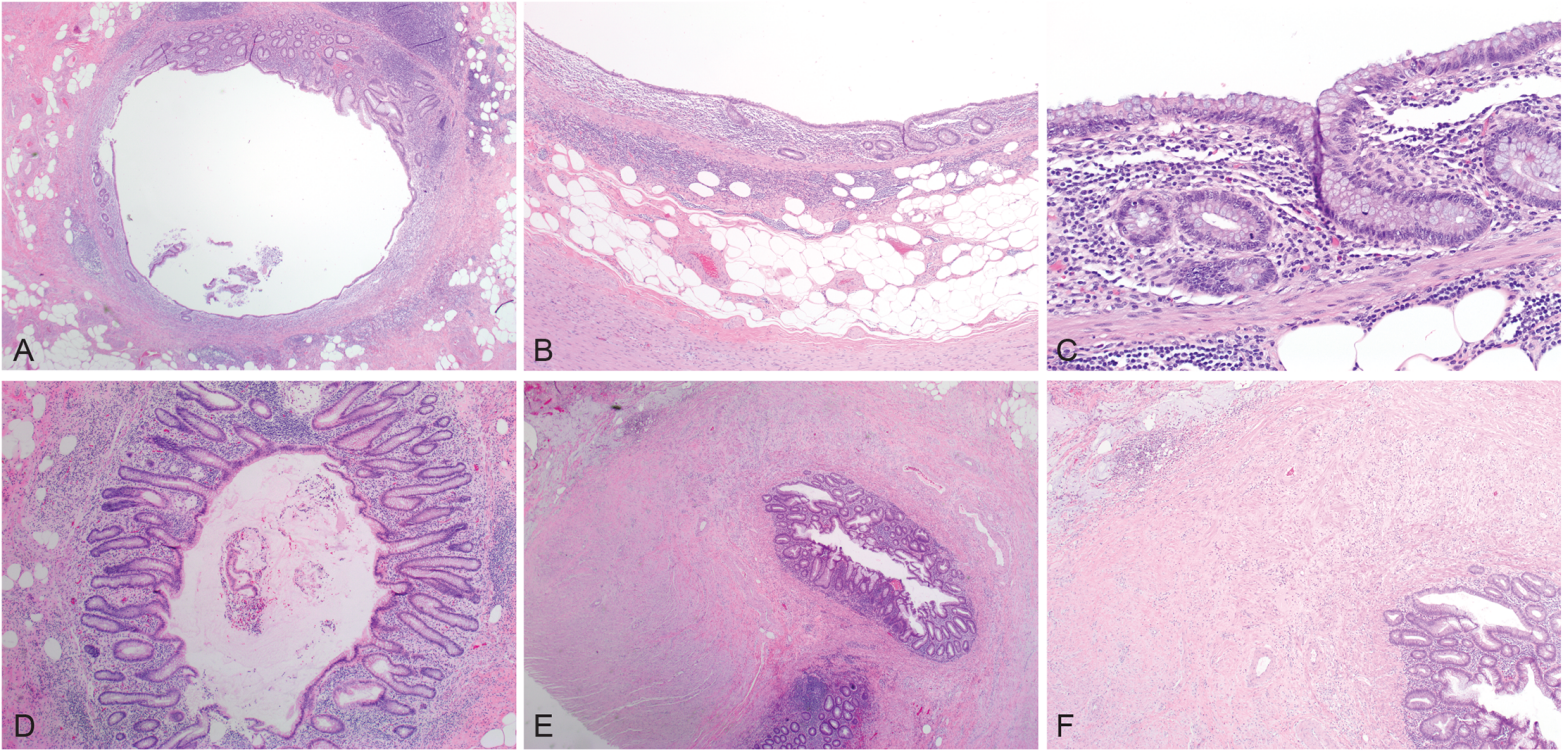
Examples of discordant simple non-neoplastic mucocele cases. A and B, Appendiceal cross sections with concentric mucosal atrophy and architectural changes of the crypts. C, While decreased, the crypts are focally present within the lamina propria and no cytologic atypia is seen. D, Appendiceal cross section with crypt architectural changes indicating prior mucosal damage. E and F, Appendiceal tip with architectural changes and evidence of prior rupture, including transmural fibrosis and extra-appendiceal mucin. (hematoxylin& eosin, original magnifications x20 [A], x40x [B], 200x [C], 40x [D], 20x [E], and 40x [F]).

**Fig 3:**
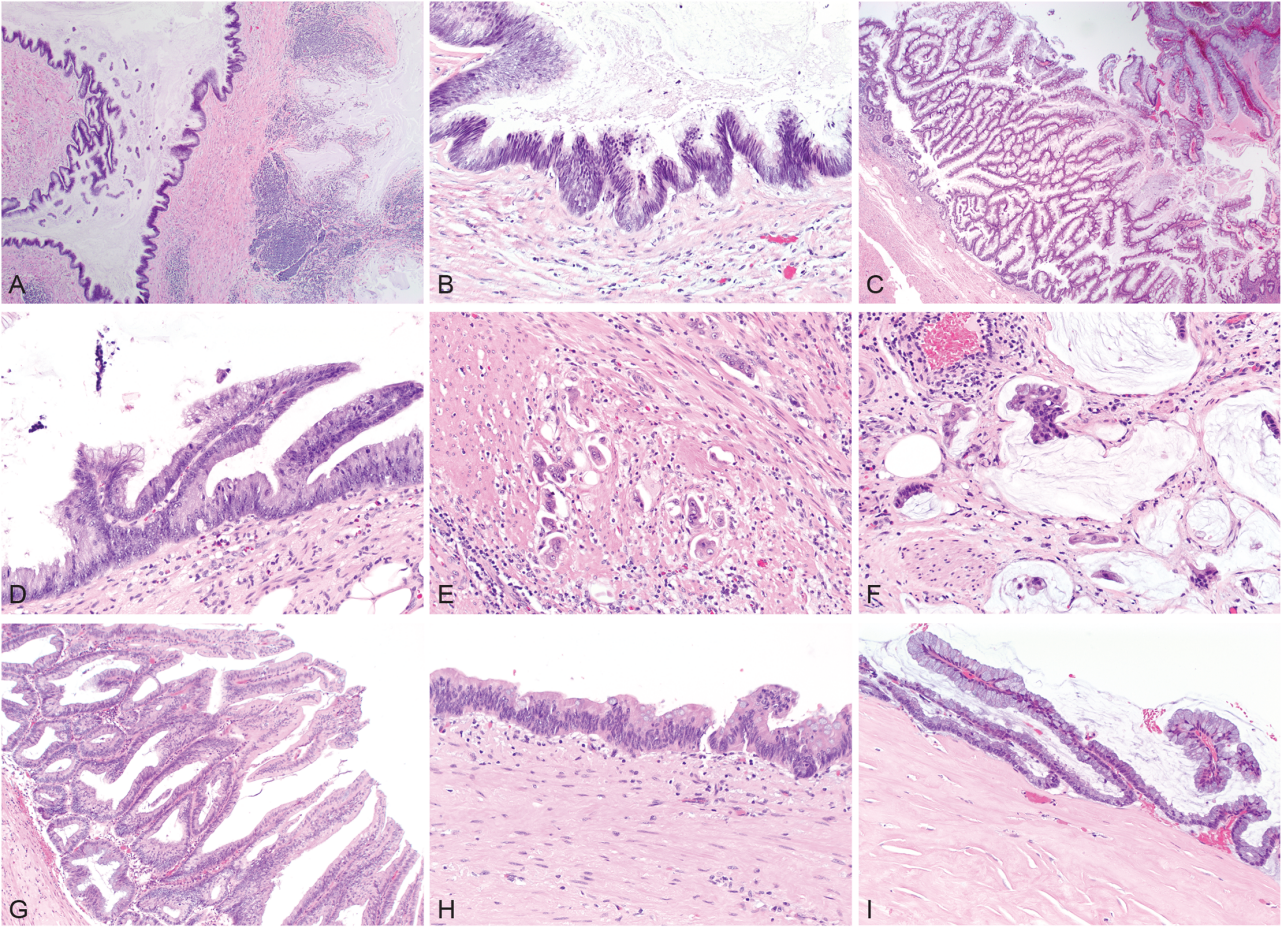
Examples of low-grade appendiceal mucinous neoplasms (LAMN) and adenocarcinoma. A, Appendiceal base with LAMN and acellular mucin. B, Cytologic atypia seen in LAMN with subjacent abnormal fibrotic stroma without intact lamina propria. C, Florid case of LAMN. D, Cytologic atypia seen in LAMN with undulating and flat profiles and abnormal underlying stroma. E, Invasive adenocarcinoma arising from LAMN and penetrating smooth muscle of the muscularis propria. F, Invasive mucinous adenocarcinoma arising from LAMN with invasion of the smooth muscle layers seen in the bottom left portion of the micrograph. G, LAMN. H, LAMN with abnormal stroma lacking an intact lamina propria. I, Peritoneal extension of LAMN which resembles the luminal neoplasm and includes a hyalinized stroma. (hematoxylin& eosin, original magnifications x40 [A], x200 [B], x40 [C], x200 [D], x400 [E], x400 [F], x200 [G], x200 [H], and x200 [I]).

For cases in which the originating pathologist rendered a diagnosis of adenocarcinoma, the reference pathologist rendered a diagnosis of adenocarcinoma (56.5%, 13 of 23), LAMN (39.1%, 9 of 23), or simple mucocele (4.3%, 1 of 23). For cases in which the originating pathologist rendered a diagnosis of LAMN, the reference pathologist rendered a diagnosis of LAMN (87.0%, 20 of 23) or simple mucocele (13.0%, 3 of 23). Conversely, when the reference pathologist rendered a diagnosis of adenocarcinoma, the originating pathologist was universally in agreement (100%, 13 of 13). When the reference pathologist rendered a diagnosis of LAMN, the originating pathologist rendered a diagnosis of adenocarcinoma (31.0%, 9 of 29) or LAMN (69.0%, 20 of 29). When the reference pathologist rendered a diagnosis of simple mucocele (non-neoplastic), the originating pathologist rendered a diagnosis of adenocarcinoma (25%, 1 of 4) or LAMN (75%, 3 of 4, Figs 2 and 3).

The differences in distributions of major diagnostic categories resulted in an overall concordance rate of 71.7% (33 of 46, Fig 1). When utilizing the secondary academic pathologist evaluation as the reference evaluation, the originating pathologists diagnosed invasive adenocarcinoma with a concordance rate of 100% (13 of 13), whereas LAMN was diagnosed with a concordance rate of 69% (20 of 29), and mucocele was diagnosed with a concordance rate of 0% (0 of 4). The overall unweighted kappa value was 0.48 (95% CI, 0.27–0.69) consistent with moderate interobserver agreement. It should be noted that in no case were there disagreements in interpretation due to under-interpretation by the originating pathologist. Therefore, all of the discordance (28.3%, 13 of 46 cases) can be attributed to over-interpretation by the originating pathologist when compared to the secondary reference pathologist.

### Diagnostic challenges for LAMN

Of the 29 LAMN cases as diagnosed by the reference pathology group, nine were originally diagnosed as adenocarcinoma (31.0%, 9 of 29). Additional potential diagnostic deficiencies were identified including discordant documentation of features needed for risk stratification (6.9%, 2 of 29) in which both such cases were documented as confined to the appendix at the originating pathology practice; whereas, the reference pathologist believed there to be extra-appendiceal neoplastic epithelium (i.e. high risk of recurrence). Several cases (31.0%, 9 of 29) lacked documentation of extra-appendiceal neoplastic epithelium by histological description, risk stratification (i.e. “high risk of recurrence” or “low risk of recurrence”), or TNM staging in the original pathology report; however, one of these cases (1 of 9) could not be categorized by the reference pathologist. Two LAMN cases (6.9%, 2 of 29) used non-standard terminology, namely “mucinous cystic neoplasm of uncertain malignant potential” and “appendiceal mucinous neoplasm with focal high-grade dysplasia” according to consensus guidelines which would advocate the use of LAMN and high-grade appendiceal mucinous neoplasm (HAMN), respectively.[3] It should be noted that advocacy for the use of HAMN for primary intraluminal appendiceal mucinous neoplasm that mimics LAMN but has high-grade cytologic features was strong, but not universal in the consensus statement (68%, 30 of 44).[3] Importantly, neither of these two cases had documentation of the presence or absence of extra-appendiceal neoplastic epithelium. Therefore, a total of 19 of 29 LAMN cases (65.5%) had potential diagnostic deficiencies when compared to the reference pathology evaluation.

## Discussion

### Discordance in primary appendiceal lesions

This study demonstrates that the overall concordance rate in primary appendiceal mucinous neoplasia is 71.7% (kappa 0.48) consistent with moderate interobserver agreement as seen in recent normative community to academic patient referral practice. Interestingly, the discordance for appendiceal neoplasia (28.3%) is quite similar to that seen when breast biopsy diagnoses were compared among pathologists (24.7%) [24]. When utilizing the secondary academic pathologist evaluation as the reference evaluation, the originating pathologists diagnosed invasive adenocarcinoma with a concordance rate of 100% (13 of 13), whereas LAMN was diagnosed with a concordance rate of 69% (20 of 29), and mucocele was diagnosed with a concordance rate of 0% (0 of 4). These latter findings may be due in part to a referral bias in which patients with malignant but not benign diagnoses as dictated by the originating pathology report are referred to an academic center for oncologic care, thus selecting for cases with malignant diagnoses. Therefore, our study is likely to be a sensitive test for over-interpretation in the community. Indeed, 31% of LAMNs (9 of 29) and 100% of mucoceles (4 of 4) in this study were over-interpreted by the originating pathologist, and over-interpretation accounted for 100% of the overall major category discordance. It should be noted that 2 cases of LAMNs had discordant risk stratification in which the originating pathologist rendered a low-risk categorization, and the secondary reference pathologist rendered a high-risk categorization; therefore, at this hierarchical level under-interpretation was identified. If this distinction were included in the overall concordance, the rate would be 67.4%, rather than 71.7%. LAMN cases lacking risk stratification (i.e. documentation of extra-appendiceal neoplastic epithelium) were considered potential diagnostic deficiencies rather than a specific diagnostic category, but appear to be an important source of reporting differences as the reference pathologist could resolve 88.9% (8 of 9) of LAMN cases initially lacking risk stratification. Equal numbers of adenocarcinoma and LAMN (as diagnosed by the originating pathologist) were referred for care, and since either diagnosis can be sufficient for referral, the referral bias may be limited, although the percentage of cases referred from each of those two categories is unknown and could differ. It is nevertheless possible that the current study may underestimate the actual rate of under-interpretation in the community; therefore, the actual overall diagnostic discordance for appendiceal lesions at large may also be underestimated.

The concordance rates in this study are compared to normative practice in a large academic referral center for appendiceal neoplasms. Neither specific training nor test sets were given to the originating or secondary pathologists to improve conformity to consensus guidelines. Common practice at our institution is in accord with current diagnostic standards and is compatible with recently published guidelines by the Peritoneal Surface Oncology Group International and the World Health Organization.[2,3] Therefore, the findings measure actual diagnostic concordance in referral practice, but may also be a reasonable approximation of discordance with current diagnostic standards.

Although the total number of LAMNs and mucoceles in the catchment community is unknown, the presence of multiple over-interpreted cases as identified in this study suggests the possibility of a relatively common systematic error occurring in the routine pathological evaluation of such cases. We found no clear patterns with respect to non-academic versus academic practices, practice size, or gastrointestinal subspecialization; however, it should be noted that additional larger studies are needed to better understand these and other parameters involved in the process of rendering complex pathological diagnoses in general, and LAMNs and related mucinous lesions in particular. Our findings do suggest an awareness of the difficulty of appendiceal mucinous neoplasia cases given that 37% of the cases (17 of 46) were shown to another pathologist at the originating practice, as compared to common peer case review rates for general quality control and assurance measures according to our experience and others that are usually ≤10% [26].

We speculate that misdiagnosis occurs, at least in part, due to apparent misconceptions regarding these lesions including the incorrect concepts that mucoceles are always neoplastic and that LAMNs are equivalent to adenocarcinomas. In addition, there appears to be confusion regarding the significance of extra-appendiceal neoplastic cells (confers high risk of recurrence). Acellular mucin must be carefully scrutinized in every section in which it occurs and the appendix specimen must be entirely submitted to exclude the presence of extra-appendiceal neoplasm. Rarely, degenerating extra-appendiceal mucinous epithelial cells can mimic histiocytes or mesothelial cells, thereby necessitating immunohistochemical evaluation for clarification (i.e. CDX2 for staining mucinous intestinal-type epithelium, CD68 for histiocytes, and calretinin, WT-1 or other markers for mesothelial cells).

### Diagnosis of primary appendiceal lesions

To further highlight diagnostic criteria and to aid in proper categorization of appendiceal neoplasia cases, we have included a checklist in Table 1. Regardless of the absence or presence of acute appendicitis, any abnormal appearing appendix with a dilated lumen, excessive mucin, or abnormal appearing epithelium or mucosa (i.e. cytological atypia, architectural changes, mucosal atrophy, etc.), should be evaluated more closely and submitted entirely for histological evaluation to exclude the presence of luminal neoplastic/dysplastic epithelium. If there is no luminal neoplastic/dysplastic epithelium after comprehensive histological evaluation, then the appendix is reactive or hyperplastic, represents a simple non-neoplastic mucocele (i.e. retention cyst), or may harbor a serrated polyp (this category includes hyperplastic polyps and sessile serrated adenomas). If definitive luminal neoplastic/dysplastic epithelium is present, then identification of destructive invasion is critical for determining whether there is invasive adenocarcinoma [2,15]. Features of destructive or infiltrative invasion have been most recently defined specifically as including tumor budding (discohesive single cells or clusters of up to 5 cells) and/or small, irregular glands typically within a desmoplastic stroma characterized by a proteoglycan-rich extracellular matrix with activated fibroblasts/myofibroblasts with vesicular nuclei.[3] Once invasive adenocarcinoma has been established, then subclassifying as well-, moderately-, or poorly-differentiated is straightforward and similar to other sites in the gastrointestinal tract; adenocarcinomas with <50% mucin (or signet ring cells) should be diagnosed as adenocarcinoma with mucinous (or signet ring cell) features; whereas, adenocarcinomas with >50% mucin (or signet ring cells) should be diagnosed as mucinous (or signet ring cell) adenocarcinoma.[2] Rarely, appendiceal endometriosis involved by intestinal metaplasia can occur and mimic adenocarcinoma.[27,28]

**Table 1:** Proposed checklist for appendiceal neoplasia.

**Appendix Entirely Submitted**	
	___ Yes, entirely submitted
	___ No, representative sections only
	___ Unknown
**Definitive Luminal Neoplastic Epithelium**	
	___ Present
	___ Not identified
	___ Cannot be determined
**Loss of Lamina Propria/Muscularis Propria and Stromal Hyalinization**	
	___ Present
	___ Not identified
	___ Cannot be determined
**Extra-appendiceal Mucin**	
	___ Present
	___ Not identified
	___ Cannot be determined
Extra-appendiceal Neoplastic Epithelium*	
	___ Present
	___ Not identified
	___ Cannot be determined
Destructive or Infiltrative Invasion/Tumor Budding**	
	___ Present
	___ Not identified
	___ Cannot be determined
**Surgical Evaluation for Peritoneal Disease**	
	___ Yes, peritoneal disease present
	___ Yes, peritoneal disease absent
	___ Evaluation not performed or incomplete
	___ Unknown

* If present, confers high risk of recurrence in LAMN

** Small, irregular glands in a desmoplastic stroma and/or discohesive single cells or clusters of up to 5 cells; if present, diagnostic of adenocarcinoma

Another key problem area is differentiating neoplastic/dysplastic lesions that lack overt destructive invasion. These lesions include adenomas of various sorts that mimic their colorectal counterparts (i.e. tubular adenoma, tubulovillous adenoma, traditional serrated adenoma, sessile serrated adenoma with conventional/cytological dysplasia) and LAMN. The key discriminator between these entities is the presence of so-called pushing invasion present in LAMNs which manifests itself as obliteration of the lamina propria and/or muscularis mucosae (may have loss of the normal mucosa associated lymphoid tissue) and fibrosis and hyalinization of the underlying stroma (generally submucosa or deeper levels of the appendiceal wall). There can also be associated luminal dilatation, diverticula, and sometimes perforation and rupture of the appendix, although these features are not specific to LAMN and can be seen in non-neoplastic retention cysts. In contrast, colorectal adenomas exhibit expected architecture as seen in the colon and rectum, and have intact lamina propria and muscularis mucosae, except in cases in which the neoplasm has already progressed to a frankly invasive adenocarcinoma. Serrated polyps of the appendix have serrated luminal profiles often with basal dilatation as can be seen in sessile serrated adenomas of the colon and rectum. In general, these lack overt cytological (also called conventional) dysplasia. Cases of serrated polyp with extensive cytological dysplasia may be difficult to distinguish, but should have serrated profiles and intact lamina propria and muscularis mucosae. Moreover, serrated polyp may occasionally have mutated BRAF which is detectable by molecular or immunohistochemical methods.[29,30] Other conventional adenomas of the appendix should be similar to their colorectal counterparts, and lack pushing invasion having an intact lamina propria and muscularis mucosae. In villform lesions, care should be taken to evaluate the basal architecture and stroma to determine whether or not LAMN is a diagnostic possibility. One study suggests combining conventional adenomas and LAMN into a single diagnostic category in part because of their high rates of KRAS and low rates of BRAF mutation; however, their capacity for peritoneal dissemination differs. [29]

Once a diagnosis of LAMN has been made, then determination of the extent of the neoplasm is critical–specifically whether there is extra-appendiceal neoplastic epithelium. To fully exclude the presence of extra-appendiceal neoplastic epithelium, the entire appendix must be submitted for histologic evaluation. The extra-appendiceal neoplastic epithelium can closely line the peritoneal surface or can be organized as strips and islands of bland-appearing neoplastic epithelium floating in large pools of mucin outside the wall. Difficulty arises in areas of perforation without clearly externalized neoplastic epithelium, because rupture or perforation itself is a necessary, but not a sufficient, event to confer a high-risk of recurrence.[15] In these rare cases, confident documentation of the presence or absence of extra-appendiceal neoplastic epithelium may not be possible on histological assessment, and therefore close clinical follow-up would be warranted. Indeed, in the current study one such case was encountered (3.4%, 1 of 29 LAMN cases) in which neither the originating or secondary pathologist determined a risk of recurrence. In contrast to extra-appendiceal neoplastic cells, extra-appendiceal acellular mucin does not confer high risk, but should be cause for carefully scrutinizing the specimen for the presence of extra-appendiceal neoplastic cells. If a LAMN is completely confined by the appendix (i.e. only present within the appendiceal lumen without evidence of perforation or extra-appendiceal mucin after complete histological evaluation) and there is no gross evidence of peritoneal disease at the time of surgery, then there is a negligible risk of recurrence as previously published studies indicate that such patients remain disease free upon follow up (0% recurrence, 0/75 patients).[14,15,18,19] Nevertheless, clinical follow-up may be warranted in certain patients, and additional larger studies are needed to help further establish the precise risk of recurrence in this specific scenario.

Appendiceal mucocele is a descriptive macroscopic or gross term used for a dilated appendix filled with mucin. In our opinion, despite continued usage, appendiceal mucocele should not be used to connote neoplasia. In this study, the term simple mucocele has been used to refer to a non-neoplastic “mucous retention cyst”. It has been observed that these non-neoplastic mucoceles generally have a small diameter and rarely exceed 1.5–2.0 cm in diameter; whereas, the vast majority of cases with larger diameters are indeed neoplastic.[13,31] On histology, simple mucoceles lack ctyologic atypia and neoplastic epithelium, and have at least partially intact lamina propria including crypt profiles.

### Staging of appendiceal neoplasia

A diagnosis of adenocarcinoma requires evidence of destructive tissue invasion in the form of irregular glands with desmoplasia, or overtly invasive individual cells (including signet ring cells).[15] Despite LAMN lacking destructive tissue invasion and having a much better prognosis as compared to conventional invasive adenocarcinoma, they have been placed in the adenocarcinoma category by both the WHO and the American Joint Committee on Cancer (AJCC) in 2010.[2,32] The WHO acknowledges LAMN as a distinct nosological entity separate from mucinous adenocarcinoma and signet ring cell carcinoma; however, the AJCC accommodates differences in outcomes only by distinguishing mucinous versus non-mucinous adenocarcinomas.[2,32] The limitations of these classification systems becomes clear when considering an truly invasive adenocarcinoma that arises from precursor LAMN. Indeed, 23.1% of the invasive adenocarcinoma cases in this series arose from a background of LAMN. Another confounder is that LAMN can have quite extensive surface disease (without invasion) of the peritoneum, involve the ovaries, uterus, and even the endometrial surface presumably via fallopian tube transit.[33,34] Despite this, at our institutions, we currently continue to utilize both carcinoma pathological staging (TNM, as per AJCC) and “risk stratification” to emphasize the extent of tumor and to aid in determination of appropriate clinical follow up; however, it should be reiterated that the presence or absence of extra-appendiceal should always be documented in some manner in cases of LAMN.[15,16,32]

## Conclusions

LAMN and their peritoneal dissemination remain a difficult area for appropriate pathological classification. This study demonstrates a moderate diagnostic agreement for the diagnosis of primary appendiceal mucinous neoplasia in normative pathology and patient referral practice. Published consensus guidelines may facilitate clarification of classification and improvement of diagnostic concordance; however, clarified algorithmic diagnostic approaches based closely on the anatomy and biological potential of these neoplasms are needed. Because of the inherent difficulties of these cases, many with borderline malignant potential and disrupted anatomy, we recommend consideration of expert secondary pathological review at large academic referral centers that frequently encounter these lesions. Further studies are needed to help clarify clinical outcomes and improve current diagnostic evaluation in part by incorporating emerging biomarkers will the goal of providing improved prognostic and therapeutic information for patient care regarding this disease.

## Supporting information

S1 Table Referred primary appendiceal lesion case characteristics. (PDF)
