# Supplementary material for: Overinterpretation is common in pathological diagnosis of appendix cancer during patient referral for oncologic care
Source: PLoS One. 2017 Jun 7;12(6):e0179216. doi: 10.1371/journal.pone.0179216 (PMC5462425; doi:10.1371/journal.pone.0179216)
Supplement: S1 Table — (PDF) [file pone.0179216.s001.pdf]

	Reference Institution					Originating Institution											
Case #	Ref Diagnosis	Pathologic Stage	Explicit Risk or Cells (High or Low for LAMN)	Reviewed by GI specialist? (Y/N)	Reviewed by GI specialist with interest in appendix (Y/N)?	Outside Diagnosis	Pathologic Stage	Explicit Risk or Cells (High or Low for LAMN)	Margin	Procedure	Practice size (# of anatomic pathologists)	Case shown to another pathologist? (Yes/No)	# Add'l Pathologists	Was signing out pathologist GI Fellowship trained? (Yes/No)	Practice setting (Academic/Non-academic)	Approximate annual surgical case volume; in thousands	Major Category Discrepancy (Yes/No)
1	LAMN	pT4a Nx	Not specified	Y	Y	Adenocarcinoma arising in mucinous cystadenoma	pT4a MX N1a	Not specified	Negative	Appendectomy	5	No	0	No	Non-academic	7	Yes
2	LAMN	pT4a Nx	High	Y	Y	LAMN with probable rupture	Not specified	Not specified	Negative	Appendectomy	4	No	0	No	Non-academic	4	No
3	LAMN	pT4a Nx	Not specified	Y	N	LAMN	specified	High	Negative	Appendectomy	8	Yes	0	No	Academic	2	No
4	LAMN	pT4a N0 M1a	High	Y	Y	LAMN	Not specified	High	Positive (proximal hemicolectomy margin)	Right hemicolectomy and segmental resection	7	No	0	No	Non-academic	20	No
5	LAMN	pT4a NX	High	Y	Y	LAMN with extrappendiceal involvement	pT4a NX	High	Negative	Appendectomy	29	Yes	1	Yes	Academic	20	No
6	LAMN	pT3 vs pT4a NX	Not specified	Y	N	LAMN	Not specified	Not specified	Negative	Appendectomy	Unknown	No	0	No	Non-academic	39	No, risk lacking (both)
7	LAMN	pT4a NX	Not specified	Y	N	LAMN mucinous epithelium on external surface	Not specified	Not specified	Negative	Appendectomy	15	No	0	No	Non-academic	63	No
8	LAMN	pTis NX	Not specified	Y	N	LAMN with external acellular mucin	Not specified	Not specified	Negative	Appendectomy	22	Yes	0	Yes	Non-academic	5	No
9	LAMN	pTis NX	Low	Y	N	LAMN with external acellular mucin	Not specified	Not specified	Negative	Appendectomy	10	Yes	0	No	Non-academic	34	No
10	Moderately differentiated adenocarcinoma with mucinous	pT3NoM1a	N/A	Y	Y	Moderate to poorly differentiated adenocarcinoma with mucinous features	pT4b N0 M1a	Not applicable	Negative	Right hemicolectomy	8	No	0	No	Non-academic	32	No
11	LAMN	Not specified	Not specified	Y	Y	Invasive mucinous adenocarcinoma	Not specified	Not specified	Not specified	Appendectomy and right hemicolectomy	Unknown	No	0	No	Non-academic	6	Yes
12	Adenocarcinoma ex GCC	pT3 NX	N/A	Y	N	Poorly differentiated carcinoma ex goblet cell carcinoid	pT3 NX	N/A	Positive	Appendectomy	12	No	0	No	Non-academic	5	No
13	LAMN	Not specified	High	Y	Y	LAMN with perforation	Not specified	Not specified	Negative	Appendectomy	5	No	0	No	Non-academic	6	No
14	LAMN	pT4aNXM1a	Not specified	Y	N	Mucinous adenocarcinoma	Not specified	Not specified	Negative	Appendectomy	Unknown	No	0	No	Non-academic	29	Yes
15	LAMN	pT4a NX M1a	High	Y	Y	Low grade mucinous cystadenocarcinoma	pT4aNXM1a	Not specified	Negative	Appendectomy	7	No	0	No	Non-academic	37	Yes, terminology
16	Adenocarcinoma ex LAMN	pT4a NX	N/A	Y	Y	Mucinous adenocarcinoma	pT4a NX	N/A	Negative	Appendectomy	10	No	0	Yes	Non-academic	6	No
17	Adenocarcinoma with mucinous features	pT4a N0 M1a	N/A	N	N	Adenocarcinoma with mucinous features	pT4a N1c M1	N/A	Positive	Right hemicolectomy	4	No	0	No	Non-academic	5	No
18	Adenocarcinoma ex GCC	pT4a NX	N/A	Y	Y	Adenocarcinoma ex GCC	pT4a NX	N/A	Positive (mesenteric)	Appendectomy	3	No	0	No	Non-academic	18	No
19	LAMN	pT4a N0 M1a	Not specified	Y	Y	LAMN	pT4a N0 M1a	Not specified	Positive	Ileocecectomy	6	Yes	1	No	Non-academic	6	No
20	LAMN	pT4a NX	High	Y	Y	Well differentiated/low grade mucinous adenocarcinoma	Not specified	Not specified	Not specified	Appendectomy	39	Yes	3	Yes	Academic	56	Yes
21	Adenocarcinoma	pT4a N1 M1b	N/A	Y	Y	Adenocarcinoma with signet ring cell features	pT4 N1 M1b	N/A	Negative	Right hemicolectomy	19	Yes	1	No	Non-academic	5	No
22	Adenocarcinoma ex GCC	pT3 NX	N/A	N	N	Adenocarcinoma ex GCC	pT3 NX	N/A	Positive	Appendectomy	23	Yes	1	Yes	Non-academic	64	No
23	LAMN	Not specified	Low	Y	Y	LAMN	Not specified	Not specified	Not specified	Appendectomy	5	No	0	Yes	Non-academic	6	No, risk lacking
24	LAMN	Not specified	High	Y	Y	LAMN	Not specified	Not specified	Not specified	Appendectomy	19	No	0	No	Non-academic	5	No, risk lacking
25	LAMN	Not specified	Low	Y	N	Mucinous cystic neoplasm of uncertain malignant potential	Not specified	Not specified	Not specified	Appendectomy	8	Yes	1	No	Non-academic	32	No, risk lacking, terminology
26	Ruptured mucocele	Not applicable	N/A	Y	Y	LAMN	Not specified	Not specified	Not specified	Appendectomy	6	No	0	No	Non-academic	1	Yes
27	LAMN	Not specified	Not specified	Y	Y	Mucinous adenocarcinoma	pT4a NX	Not applicable	Positive	Appendectomy	3	No	0	No	Non-academic	9	Yes
28	LAMN	pT2 NX	Low	Y	Y	Poorly differentiated adenocarcinoma ex goblet cell carcinoid	Not specified	Not applicable	Not specified	Appendectomy and cecectomy	6	Yes	1	No	Non-academic	1	Yes
29	Adenocarcinoma, moderately-to-poorly differentiated, arising from LAMN	pT3 N0	N/A	Y	Y	Adenocarcinoma, well differentiated, arising from villous adenoma	pT4a N0 M1a	N/A	Negative	Appendectomy	5	No	0	No	Non-academic	26	No
30	LAMN	pT4a N0 M1a	High	Y	Y	Appendiceal mucinous neoplasm with focal high-grade dysplasia	Not specified	Not applicable	Negative	Appendectomy, cecectomy, and pelvic mass resection	8	No	0	No	Non-academic	10	No, risk lacking, terminology
31	LAMN	pT3 NX	High	Y	Y	Low grade mucinous adenocarcinoma	Not specified	Not specified	Negative	Appendectomy	6	No	0	No	Non-academic	36	Yes
32	Ruptured mucocele	N/A	N/A	Y	Y	LAMN	Not specified	Not specified	Negative	Appendectomy	6	No	0	No	Non-academic	36	Yes
33	Ruptured mucocele	N/A	N/A	Y	Y	Appendiceal mucinous neoplasm with high risk of recurrence	Not specified	High	Negative	Appendectomy	6	No	0	No	Non-academic	36	Yes
34	LAMN	pT4a N0	High	Y	Y	LAMN	Not specified	Not specified	Negative	Appendectomy	13	Yes	0	No	Non-academic	52	No, risk lacking
35	Adenocarcinoma ex GCC	pT2 NX	N/A	Y	Y	Goblet cell neuroendocrine neoplasm	pT3 NX	N/A	Negative	Appendectomy	3	Yes	1	No	Non-academic	6	No
36	Ruptured mucocele	N/A	N/A	Y	Y	Mucinous adenocarcinoma	pT3 N0	High	Negative	Appendectomy	9	Yes	1	No	Non-academic	Unknown	Yes
37	Adenocarcinoma ex LAMN	pT4a NX	N/A	Y	Y	Well differentiated adenocarcinoma with mucinous features	pT3 NX	N/A	Negative	Appendectomy with partial cecectomy	1	No	0	No	Non-academic	3	No
38	LAMN	pT4a N0	Not specified	N	N	LAMN	pT3 N0	Not specified	Negative	Appendectomy	17	No	0	No	Non-academic	58	No, risk difference
39	LAMN	Not specified	High	Y	Y	Low grade appendiceal mucinous adenocarcinoma	Not specified	High	Negative	Appendectomy	5	Yes	1	No	Non-academic	25	Yes
40	Adenocarcinoma	pT4 NX	N/A	Y	Y	Poorly differentiated adenocarcinoma with prominent signet-ring features	pT4 NX M1	N/A	Positive	Appendectomy	4	No	0	No	Non-academic	19	No
41	Adenocarcinoma with mucinous features	pT4a NX M1a	N/A	Y	Y	Well differentiated mucinous adenocarcinoma	pT4a NX M1	N/A	Positive	Appendectomy, hysterectomy, bilateral salpingo-oophorectomy, splenectomy, omentectomy	7	No	0	No	Non-academic	18	No
42	LAMN	Not specified	High	Y	Y	LAMN	Not specified	Low	Negative	Appendectomy	20	Unknown	0	Yes	Non-academic	60	No, risk difference
43	LAMN	pT4a NX M1a	Not specified	N	N	LAMN	Not specified	Not specified	Not specified	Appendectomy	8	Yes	1	No	Non-academic	32	No, risk lacking
44	LAMN	pT3 NX	Not specified	Y	Y	LAMN	Not specified	Not specified	Positive	Appendectomy	10	Yes	0	No	Non-academic	Unknown	No, risk lacking
45	LAMN	Not specified	Low	Y	Y	LAMN	Not specified	Not specified	Not specified	Appendectomy and right hemicolectomy	26	Yes	1	No	Non-academic	13	No, risk lacking
46	Adenocarcinoma with focal mucinous features	pT3 NX	N/A	Y	Y	Moderate to poorly differentiated adenocarcinoma with mucin production	Not specified	N/A	Appendiceal negative	Appendectomy	6	No	0	No	Non-academic	36	No
